# Beneficial and Safety Properties of a Bacteriocinogenic and Putative Probiotic *Latilactobacillus sakei* subsp. *sakei* 2a Strain

**DOI:** 10.3390/foods13233770

**Published:** 2024-11-25

**Authors:** Tatiana Alexandrovna Lipilkina, Cristhian Xu, Matheus de Souza Barbosa, Valentina Nikolaevna Khramova, Sergei K. Shebeko, Alexey M. Ermakov, Iskra Vitanova Ivanova, Svetoslav Dimitrov Todorov

**Affiliations:** 1 ProBacLab, Laboratório de Microbiologia de Alimentos, Departamento de Alimentos e Nutrição Experimental, Food Research Center, Faculdade de Ciências Farmacêuticas, Universidade de São Paulo, São Paulo 05508-000, Brazil; tterpitskaya@gmail.com (T.A.L.); cristhian.xu@usp.br (C.X.); matheusdesb@hotmail.com (M.d.S.B.); iskrai3@yahoo.com (I.V.I.); 2 Faculty of Bioengineering and Veterinary Medicine, Don State Technical University, Gagarina Sq., 1, Rostov-on-Don 344002, Russia; shebeko_sk@mail.ru (S.K.S.); amermakov@ya.ru (A.M.E.); 3 Department of Food Production Technology, Volgograd State Technical University, V.I. Lenin Avenue, 28, Volgograd 400005, Russia; hramova_vn@mail.ru; 4 Department of General and Applied Microbiology, Faculty of Biology, Sofia University St. Kliment Ohridski, 8 Dragan Tzankov Blvd., 1164 Sofia, Bulgaria; 5 CISAS-Center for Research and Development in Agrifood Systems and Sustainability, Instituto Politécnico de Viana do Castelo, 4900-347 Viana do Castelo, Portugal

**Keywords:** *Latilactobacillus sakei*, starter cultures, probiotics, bacteriocins, safety, virulence factors, antibiotic resistance

## Abstract

This work aimed to evaluate some of the probiotic features and safety of the bacteriocin-producing *Latilactobacillus sakei* subsp. *sakei* 2a. The effect of selected commercial drugs from different generic groups and antibiotics on the growth of *Ltb. sakei* subsp. *sakei* 2a was also determined. The presence of virulence factors was determined based on PCR with total DNA from *Ltb. sakei* subsp. *sakei* 2a. Good growth of *Ltb. sakei* subsp. *sakei* 2a was recorded in MRS broth supplemented with 0.2% or 0.4% oxbile or in MRS broth adjusted to a pH from 5.0–9.0. Auto-aggregation of *Ltb. sakei* subsp. *sakei* 2a was 62.59%. Different levels of co-aggregation were recorded between *Ltb. sakei* subsp. *sakei* 2a and *Enterococcus faecalis* ATCC19443, *Ltb. sakei* ATCC15521 and *Listeria monocytogenes* ScottA. Growth of *Ltb. sakei* subsp. *sakei* 2a was not inhibited by commercial drugs from different generic groups. The inhibitory effect on the growth of *Ltb. sakei* subsp. *sakei* 2a was recorded only in the presence of Arotin [selective serotonin reuptake inhibitor antidepressant] Minimal Inhibition Concentration (MIC) 1.0 mg/mL, Atlansil [Antiarrhythmic] MIC 0.625 mg/mL, Diclofenac potassium [non-steroidal anti-inflammatory drug (NSAID)] MIC 2.5 mg/mL and Spidufen [NSAID] MIC 15.0 mg/mL. Only two antibiotics tested in this study, Amoxil and Urotrobel, inhibited the growth of *Ltb. sakei* subsp. *sakei* 2a with a MIC of <0.5 mg/mL and 5.0 mg/mL, respectively. However, *Ltb. sakei* subsp. *sakei* 2a generated positive PCR results on the DNA level for *van*A (vancomycin resistance), *hyl* (hyaluronidase), *esp* (enterococcal surface protein), *ace* (adhesion of collagen) and *cil*A (cytolisin) and a high virulence profile when examined for the presence of virulence factors. It is important to underline that cytolysis has been described as a virulence and antibacterial factor.

## 1. Introduction

In the development of food science, different concepts were proposed, applied and then abandoned. With the movement of big groups of works from the rural to urban centers at the beginning of the Industrial Revolution, the need for the production, storage and distribution of food commodities, as well as change and adaptation to the needs of the society, food industry was changed and adapted to the new realities. The concentration of big groups of workers generated the need for a new way of production and conservation, and this served as a catalysator for the fundamental and applied scientific branches to respond to these needs. Progress in food processing and conservation was marked in that period. Some of the applied methods were implemented and later abandoned because the negative consequences were more than the benefits. However, chemical preservatives were winning an important role in providing reduced levels of microbial contaminates, providing extended shelf life for the food products. On the other side, chemical preservatives can result in some negative consequences. As a result, we are in the era of rewriting the food preservation rules and trying to implement the traditional way of preservation, re-evaluated via the prism of modern science, where the natural preservatives and selected beneficial starter cultures can be applied and provide healthier and safer food products [1].

In recent years, there has been growing concern over the extensive use of chemical additives in food preservation. While these additives can effectively extend shelf life and prevent spoilage, they often come with potential health risks and environmental impacts. Reducing the reliance on chemical additives is crucial for several reasons. Between them, health concerns may be a principal driving argument among consumers. Many chemical preservatives have been linked to adverse health effects, including allergies, hormonal disruptions and even carcinogenicity. By minimizing their use, we can reduce the risk of these health issues and promote safer food consumption. Moreover, the environmental impact of the application of chemical preservatives is a serious concern. The production and disposal of chemical additives can contribute to environmental pollution. Reducing their use helps decrease the chemical load on ecosystems, promoting a healthier environment. There is a growing consumer demand for natural and clean-label products. Reducing chemical additives aligns with this trend, meeting consumer preferences and potentially increasing market competitiveness [2].

As an alternative, bacteriocins offer a promising solution for food preservation. Bacteriocins are antimicrobial peptides produced by bacteria that can inhibit the growth of harmful microorganisms, and their use in food preservation presents several advantages [3]. The fact that bacteriocins are naturally occurring compounds, part of the constant metabolites produced that have already been used for millenniums by lactic acid bacteria (LAB) in food fermentation processes, making them a more acceptable option for consumers seeking natural food products. In general, bacteriocins (based on the knowledge of nisin and pediocin PA1) are generally recognized as safe (GRAS) by regulatory authorities, where levels of cytotoxicity for nisin are like that recorded for NaCl [4]. They have been used in various food products without adverse health effects, providing a safer alternative to chemical preservatives. Bacteriocins are effective against a wide range of foodborne pathogens and spoilage organisms but, at the same time, show a very specific spectrum of activity and normally do not present inhibitory effects versus starter or other beneficial microbial cultures [3]. Their use can enhance food safety and extend shelf life without the need for synthetic chemicals.

The production of bacteriocins can be more sustainable compared with chemical additives. They can be produced through fermentation processes, which are often less harmful to the environment, an argument in accordance with UN directives for sustainable industry. Reducing the use of chemical additives in food preservation is essential for health, environmental and consumer preference reasons. Bacteriocins offer a viable and effective alternative, aligning with the growing demand for natural and safe food preservation methods. Embracing these alternatives can lead to safer, more sustainable and consumer-friendly food products.

Moreover, some of the LAB can be considered regarding their probiotic potential. Probiotic properties are associated with life microbial cultures and, when applied at adequate levels, can provide health-promoting and other beneficial properties for consumers [5]. However, it is very important in this scenario that the applied probiotic microbial culture can be proven as safe, not carrying virulence factors associated with genetic determinates or antibiotic resistance [5,6]. The important point is that each strain needs to be evaluated individually for safety accordance [6].

In the last decade, an additional concept was built: the application of dead microbial cells, where the parts of the cells or already produced metabolites can express benefits as well for the consumers. The concept of postbiotics has received the attention of the research groups because the consequences of the application of life cultures (in the case of probiotics) in some cases can have negative side effects, problems with changes in microbial balance in different parts of the human and other animals’ vital systems [7]. In this regard, postbiotics can be easier evaluated regarding their beneficial effects and produced, distributed and applied [7].

In the current study, we have explored some of the beneficial and safety features of previously isolated from *linguiça* [8] and identified it as a bacteriocinogenic strain *Latilactobacillus sakei* subsp. *sakei* 2a [9]. *Ltb. sakei* is a Gram-positive, facultative anaerobic bacterium commonly associated with meat products. It plays a leading role in fermentation processes, contributing to the preservation and development of organoleptic characteristics [9,10]. Moreover, specifically the strain *Ltb. sakei* subsp. *sakei* 2a and its bacteriocin were explored, and a biopreservative and some aspects for beneficial portfolios were suggested [10]. However, the question regarding additional safety and beneficial properties is still open, and in the current paper, we aim to contribute to a better understanding of the benefits of *Ltb. sakei* subsp. *sakei* 2a.

## 2. Materials and Methods

### 2.1. Strains and Media

*Latilactobacillus sakei* subsp. *sakei* 2a was previously isolated [8] from *linguiça* and evaluated as bacteriocin/s-producing [9], and some biopreservation properties were suggested [10]. *Ltb. sakei* subsp. *sakei* 2a (this and all other lactobacilli were abbreviated according to recommendations from Todorov et al. [11]) and other LAB used in this study, such as *Ltb. sakei* ATCC15521 and *Enterococcus faecalis* ATCC19443, were cultured in MRS broth (Difco, Detroit, MI, USA), while *Listeria monocytogenes* ScottA was cultured in BHI medium (Oxoid, Hampshire, UK). All experiments were performed at 37 °C in aerobic conditions normally for 24 h. Pure cultures were stored at −80 °C in a growth medium supplemented with glycerol (15%, *v*/*v*, final concentration). Before use, all strains were incubated at least 2 times in appropriate growth conditions before being applied with further described experiments.

### 2.2. DNA Extraction and Quantification

*Ltb. sakei* subsp. *sakei* 2a was grown at 37 °C for 24 h in 20 mL MRS, and total DNA was extracted using the ZR Fungal/Bacterial Quick-DNA Fungal/Bacterial Miniprep Kit (Zymo Research, Irvine, CA, USA), according to the manufacturer’s instructions. The obtained DNA was quantified and quality assessed using a NanoDrop Spectrophotometer (Thermo Fisher, Waltham, MA, USA).

### 2.3. Biomolecular Analysis for the Evaluation Presence of Beneficial, Virulence and Antibiotic Resistance Genes

Previously obtained DNA was used for the screening for the presence of genes associated with adherence properties (*map*, *mub* and *eftu*), production of GABA (*core*), antibiotic resistance (*van*A, *van*B, *van*C, *van*D, *van*E and *van*G) and virulence factors (*gel*E, *hyl*, *asa*1, *esp*, *cyl*A, *efa*A and *ace*), biogenic amines production (*hdc*1, *hdc*2, *tdc* and *odc*). The sequence of the primers, PCR conditions and references are provided in Table 1. PCR reactions were performed on GeneAmp^®^ PCR Instrument System 9700 (Applied Biosystems, Foster City, CA, USA) at conditions recommended by the literature (Table 1). The generated amplicons were visualized in a 1.0–2.0% (*w*/*v*) agarose gel stained with SYBR^®^ Safe DNA Gel Stain (Thermo Scientific, Waltham, MA, USA) on running conditions of 100 V for 45 min and visualized with Molecular Imager^®^ GelDoc^™^ XR (Bio-Rad, Hercules, CA, USA).

### 2.4. Safety Evaluation of the Strain

In the safety evaluation of *Ltb. sakei* subsp. *sakei* 2a, hemolytic properties [17], gelatinase [18], proteolytic activity [19], sensibility to the effect of selected antibiotics and medicaments [20] and production of biogenic amines [21] were performed. The tests were performed in at least duplicate at 37 °C on two different occasions.

For the evaluation of hemolytic activity, *Ltb. sakei* subsp. *sakei* 2a was streaked on the surface of blood agar plates (Blood Agar—Laborclin—TSA *w*/5% Sheep Blood), followed by incubation at 37 °C for 48 h in aerobic conditions. Plates were observed for the formation of clear transparent zones around growing colonies, evidence for β-hemolytic activity. For α-hemolysis, green zones formed around growing colonies were considered. Moreover, the absence of halos surrounding colonies was considered as having no hemolytic activity. In the experiment, *L. monocytogenes* ScottA was used as a control for α-hemolysis [17].

In the experiment for the evaluation of gelatinase production by *Ltb. sakei* subsp. *sakei* 2a, recommendations from dos Santos et al. [18] were followed. MRS broth supplemented with 5% gelatin (Oxoid) (*w*/*v*) was used for the cultivation of *Ltb. sakei* subsp. *sakei* 2a at 37 °C for 24 h, followed by cooling for 1 h at 4 °C, and a positive result was considered when test tubes retained the liquid form after refrigeration. In the experiment, *Staphylococcus aureus* ATCC29213 was applied as positive control (gelatin hydrolysis), while an untreated medium (MRS, supplemented with 5% gelatin) was applied as negative control [18].

For the evaluation of proteolytic activity, *Ltb. sakei* subsp. *sakei* 2a was streaked on the surface of MRS agar supplemented with 10% skim milk powder (Difco) or 10% casein (Sigma-Aldrich, St. Luis, MO, USA). The plates were incubated at 37 °C for 72 h and observed for the formation of a clear zone around the colonies, evidence for the presence of proteolytic activity by examined strain [19].

Antibiotic discs (Cefar, Sao Paulo, SP, Brazil) were applied in the process for evaluation of the resistance/susceptibility of *Ltb. sakei* subsp. *sakei* 2a, listed in Table 2 according to recommendations from EFSA and some additional commonly applied antibiotics. *Ltb. sakei* subsp. *sakei* 2a was grown in MRS broth for 24 h at 37 °C and used to supplement MRS with added 2% agar in order to obtain 10^6^ CFU/mL final microbial load. The selected antibiotic discs, listed in Table 2, were placed on the surface of the prepared plate and incubated for 24 h at 37 °C following the evaluation of the formation of inhibition zones and measured (mm) to determine the strain sensitivity [20,22].

In a similar experiment, the effect of selected commercial drugs was evaluated for the inhibitory effect versus *Ltb. sakei* subsp. *sakei* 2a. The commercial drugs (Table 3) were transferred into 5 mL sterile water and homogenized for 2 min. Obtained suspensions were kept at 4 °C prior to use but no longer than 1 h. The concentration of the active ingredients of tested drugs is listed in Table 3, taking into consideration information from drug producers and the volume of the used dilutant. *Ltb. sakei* subsp. *sakei* 2a was grown in MRS broth for 24 h at 37 °C and used to supplement MRS with added 2% agar to obtain 10^6^ CFU/mL final microbial load. The selected drugs, listed in Table 3, were spotted (10 μL) on the surface of the prepared plate and incubated for 24 h at 37 °C, following evaluated for the formation of inhibition zones and measured (mm) to determine the effect of the studied drugs versus *Ltb. sakei* subsp. *sakei* 2a. Drugs presenting inhibition zones bigger than 2 mm were selected for the determination of the minimal inhibitory concentration (MIC) by preparing serial 2-fold dilutions in sterile water and 10 μL of each dilution spotted on the surface of previously prepared plated with *Ltb. sakei* subsp. *sakei* 2a, as described before. Plates were incubated for 24 h at 37 °C, evaluated for the formation of inhibition zones, and MIC determined.

### 2.5. Production of Biogenic Amines

To evaluate the potential production of biogenic amines, *Ltb. sakei* subsp. *sakei* 2a was initially cultured in MRS broth supplemented with precursors for biogenic amine production (tyrosine, ornithine, lysine and histidine, Sigma-Aldrich) at a 1:100 ratio. The inoculated tubes were incubated at 37 °C for 24 h and subsequently subcultured at least five times, following the recommendations of Bover-Cid and Holzapfel [21]. In the next step, an aliquot of ten microliters from the final broth culture of *Ltb. sakei* subsp. *sakei* 2s were individually spread onto plates containing the corresponding biogenic amine precursor. The plates were then incubated for 24 h at 37 °C. A color change in the medium from yellow to violet indicated the decarboxylation of the supplemented biogenic amine precursor, signifying the formation of biogenic amines. *Lactiplantibacillus plantarum* ST16Pa and *Escherichia coli* ATCC8739 were used as positive and negative controls, respectively.

### 2.6. Growth at Different pH and Bile Concentrations

*Ltb. sakei* subsp. *sakei* 2a was grown in MRS broth (Difco) adjusted to pH 3.0, 4.0, 5.0, 6.0, 7.0, 9.0, 11.0 and 13.0 adding 1M HCl or 1M NaOH before autoclave sterilization following recommendations from Furtado et al. [23] After autoclaving, the pH was evaluated and needed, corrected with sterile 1M HCl or 1M NaOH. In a separate experiment, the same strain was grown in MRS broth containing 0.2%, 0.4%, 0.6%, 0.8%, 1.0%, 2.0% and 3.0% (*w*/*v*) oxbile (Sigma-Aldrich) following recommendations from Furtado et al. [23] The tests were conducted in sterile flat-bottom 96-well microtiter plates (TPP, Switzerland), where each well was filled with 180 µL of the MRS with adjusted pH or bile-containing medium and inoculated with 20 µL of the culture obtained in MRS broth (Difco) (OD650 nm = 0.2) at 37 °C. For the evaluation of bacterial growth, the changes in optical density were recorded at 650 nm every hour for 12 h using a microtiter plate reader (Thermo Fisher). Cultures grown in MRS broth (Difco) served as control. Experiments were performed in triplicates.

### 2.7. Auto-Aggregation and Co-Aggregation

In the evaluation of the aggregation (auto- and co-aggregation) properties, suggestions from Pingitore et al. [24] were adapted for current experiments. *Ltb. sakei* subsp. *sakei* 2a was evaluated for his auto-aggregation and co-aggregation properties combined with *L. monocytogenes* ScottA, *Ltb. sakei* ATCC15521 and *E. faecalis* ATCC19433. Bacterial cultures were grown in 20 mL MRS broth or BHI (Difco) for 24 h at 37 °C in aerobic conditions. The cells were harvested by centrifugation (8000× *g*, 10 min, 4 °C), and obtained cells were washed twice and resuspended in sterile saline (0.85% NaCl, *w*/*v*) to a density of the suspension, corresponding to OD660 nm = 0.3. The obtained cell suspensions were incubated for 1 h at 37 °C, centrifugated for 2 min at 300× *g*, and the upper phase was evaluated at 660 nm spectrophotometrically. The efficiency of auto-aggregation was calculated according to Pingitore et al. [24], where % of auto-aggregation was calculated as [(OD0 − OD60)/OD0] × 100, where OD0 corresponded to the initial OD of the suspension and OD60 to OD after incubation and centrifugation as indicated before, measured at 660 nm.

In experiment for the determination of co-aggregation properties, equal volumes of paired cell suspensions, prepared as described before and after incubation for 60 min at 37 °C of evaluated strain (*Ltb. sakei* subsp. *sakei* 2a) and co-aggregation partners (*L. monocytogenes* ScottA, *Ltb. sakei* ATCC15521 and *E. faecalis* ATCC19433) were evaluated. OD measured at 660 nm was recorded for the upper phase after separation by centrifugation for 2 min at 300× *g*, as described for the auto-aggregation experiment [24] and levels of co-aggregation calculated [(OD0 − OD60)/OD0] × 100, where OD0 correspond to the initial OD of the suspension and OD60 to OD after incubation and centrifugation as indicated before, measured at 660 nm. The experiments were conducted twice, each time in triplicate [24].

### 2.8. Role of Prebiotics on the Bacterial Growth and Production of Bacteriocins

For the evaluation of the effect of fructooligosaccharides (FOSs) on bacterial growth and production of bacteriocin by *Ltb. sakei* subsp. *sakei* 2a, a modified MRS broth was prepared, where glucose was replaced with the same level (20 g/L) FOS. MRS (with glucose) served as a control. *Ltb. sakei* subsp. *sakei* 2a was used to inoculate 100 mL of both media (MRS-FOS and MRS-glucose) with 2% inoculum and cultured at 37 °C for 24 h. After 8, 12 and 24 h of growth, aliquots were obtained and evaluated for the bacterial growth (at 600 nm), pH and production of bacteriocin against *L. monocytogenes* ScottA. For the determination of levels of produced bacteriocin, recommendations from Fugaban et al. [20] were followed. The supernatant was obtained after centrifugation (8000× *g*, 10 min at 4 °C), the pH was corrected to 5.5–6.5 with 1M NaOH and heat-treated for 10 min at 80 °C [20]. For the evaluation of bacteriocin production, plates with BHI supplemented with 1% agar and inoculated with *L. monocytogenes* ScottA at a final concentration of 10^6^ CFU/mL were prepared. Previously prepared with corrected pH and heat-treated cell-free supernatant were 2-fold serially diluted with 25 mM sodium phosphate buffer (pH 6.5) and 10 μL of each dilution spotted on previously prepared plates with incorporated test microorganisms [20]. Plates were incubated at 37 °C for 24 h and observed for the inhibition zones. Positives were considered inhibitions larger than a diameter of 2 mm. The bacteriocin activity was expressed as AU/mL, where was taken in consideration the type of dilutions, the volume of spotted antimicrobial material, and the number of most diluted cell-free supernatants represented an inhibitory effect [20].

## 3. Results and Discussion

### 3.1. Biomolecular Analysis for the Evaluation Presence of Beneficial, Virulence, Biogenic Amines and Antibiotic Resistance Genes

The DNA from *Ltb. sakei* subsp. *sakei* 2a generated positive results for the presence of genes associated with adherence properties (*map, mub* and *eftu*) and production of GABA (*core*) (Table 1). The presence of adherence proteins is a positive feature for the strains with probiotic properties because they can promote adherence to the gut epithelium. For the probiotics to promote the stimulation of the immune system or be involved in the production of antimicrobial metabolites, colonization of the gut is essential. Thus, the presence of the adherence proteins can be considered beneficial for the *Ltb. sakei* subsp. *sakei* 2a. The presence of genes encoding adherence proteins was previously reported for different lactobacilli [25,26]. Adherence proteins have an essential role in the effectiveness of probiotic strains. These proteins promote probiotics to adhere to the intestinal epithelial cells, essential for their colonization properties and further persistence. By adhering to the gut epithelium, probiotics can enhance the intestinal barrier function and modulate the immune system and, as a consequence, can help prevent the invasion of pathogens and maintain gut health [27]. Moreover, adherence proteins allow probiotics to compete with pathogenic bacteria for binding sites on the intestinal cells, or when probiotics can produce antimicrobial substances, adherence to the gut epithelium can enhance the antimicrobial properties and reduce it for some pathogens [28]. Processes of interaction with host cells are directly related to the presence of adherence proteins on the surface of probiotic cells. Indeed, this can lead to beneficial effects such as the modulation of inflammatory responses and the promotion of gut health. For instance, studies have shown that the surface layer proteins (SLPs) of certain lactobacilli are critical for their adhesion to intestinal cells and their subsequent probiotic effects [27].

Gamma-aminobutyric acid (GABA) is an interesting metabolite that has won the attention of the scientific community in the last decade due to its various physiological and therapeutic roles. GABA is a principal inhibitory neurotransmitter in the central nervous system. His physiological contribution is related to the reduction of neuronal excitability, which can alleviate anxiety and promote relaxation [29]. Moreover, probiotic strains that can produce GABA may contribute to the gut–brain axis and, as a consequence, potentially improve mental health. It was reported that some GABA-producing lactobacilli and bifidobacteria could be involved in managing stress and anxiety when applied as probiotics in animal models [30]. It was suggested that GABA-producing probiotics can enhance gut health by improving the intestinal barrier function. They increase the levels of tight junction proteins, which help maintain the integrity of the gut lining and prevent leaky gut syndrome [31]. Immune modulation was suggested for GABA. Probiotics that produce GABA can contribute to the regulation of immune responses and even potentially reduce inflammation and enhance overall immune function [29]. GABA can also play a role in metabolic regulation and has been associated with the regulation of blood pressure and glucose levels, which can be beneficial for managing conditions like hypertension and diabetes [29]. Moreover, GABA-producing probiotics can positively influence the gut microbiota composition. They can increase the abundance of beneficial bacteria while reducing the levels of pathogenic bacteria, contributing to a healthier gut environment [31]. A GABA producer, *Limosilactobacillus fermentum* L18, was shown to improve the gut epithelial barrier and modulate the gut microbiota [31].

Moreover, from six different genes related to vancomycin resistance, positive results were observed only for *van*A. In general, antibiotic resistance in probiotic strains is a complex and significant topic, particularly in the context of both probiotic efficacy and safety. From safety issues, the presence of antibiotic resistance genes in the probiotic strains was always considered a hazard because concerns for the spread of antibiotic resistance to other (beneficial or pathogenic) bacterial species were considered high risk and a potential scenario taking into consideration horizontal gene transfer [32]. However, Suvorov [32] pointed to several biological limitations of those processes and relatively low-risk levels for such processes to occur in biological systems. According to the regulatory framework, the presence of antibiotic resistance is considered as negative feature for the probiotic strains [33]. Taking into consideration the physiological properties of the probiotics and the fact that, in several scenarios, they will be applied in combination with antibiotics, a moderate resistance to some antibiotics can be considered even as beneficial feature because it will give an opportunity for probiotics to survive and perform his beneficial properties when applied as therapeutical agents in combination with antibiotics. However, this is a hypothesis that needs further discussion and investigation. Probiotics that are vancomycin-resistant can survive antibiotic treatments that include vancomycin. Moreover, most of the lactobacilli are resistance to vancomycin associated with intrinsic resistance [34]. Intrinsic resistance, which is naturally present in some probiotic strains, is generally considered safer because it is less likely to be transferred to other bacteria. Acquired resistance, on the other hand, often involves mobile genetic elements that can be transferred between bacteria, posing a higher risk [35]. However, enterococci resistant to vancomycin are considered as serious health hazard [36]. This is particularly useful in restoring and maintaining gut microbiota during and after antibiotic therapy, as these probiotics can continue to exert their beneficial effects without being eliminated by the antibiotic [37].

After antibiotic treatment, the gut microbiota can be significantly disrupted. Vancomycin-resistant probiotics can help in recolonizing the gut with beneficial bacteria, thereby aiding in the restoration of a healthy microbiota balance [38]. While vancomycin resistance can be beneficial for the reasons mentioned above, it also raises safety concerns. The primary concern is the potential transfer of resistance genes to pathogenic bacteria, which could lead to the development of vancomycin-resistant pathogens. This is particularly concerning because vancomycin is often used as a last-resort antibiotic for treating serious infections [37].

Due to the potential risks associated with antibiotic resistance, regulatory bodies like the EFSA require a thorough evaluation of antibiotic resistance determinants in probiotic strains before they can be approved for use. This ensures that the benefits of using such probiotics outweigh the risks [37].

Regarding some virulence factors, positive PCR reactions were recorded for the presence of *hyl*, *esp*, *cyl*A and ace (Table 1), genes related to the functionality and safety of probiotic strains, as well as their interactions with the host. Hyaluronidase, encoded by *hyl*, is an enzyme that breaks down hyaluronic acid, a major component of the extracellular matrix in tissues, and, as a consequence, can facilitate bacterial spread through tissues. While hyaluronidase activity can be beneficial in breaking down biofilms and enhancing probiotic colonization, it also poses a risk of tissue damage and inflammation if not properly regulated [39].

The *esp* gene encodes a surface protein that aids in the adhesion of bacteria to host cells and biofilm formation. This gene is crucial for the colonization and persistence of probiotics in the gut. However, its presence is also associated with increased virulence in pathogenic strains, making it a double-edged sword [39].

Cytolysin is a toxin that can lyse red blood cells and other cell types. It is encoded by the *cyl*A gene and is part of a larger operon involved in its production and regulation. While cytolysin can help probiotics outcompete pathogenic bacteria by killing them, it also poses a significant risk of damaging host tissues and contributing to pathogenicity [39].

The *ace* gene encodes a protein that facilitates the adhesion of bacteria to collagen, a structural protein in connective tissues. This adhesion capability is important for the colonization and persistence of probiotics in the gut. However, similar to *esp*, it is also associated with virulence in pathogenic strains [40].

The presence of these genes in probiotic strains must be carefully evaluated to balance their beneficial effects with potential risks. For instance, while these genes can enhance the colonization and competitive abilities of probiotics, they also carry the risk of contributing to pathogenicity if transferred to pathogens in similar processes previously discussed for antibiotic-resistant genes [39].

Detection of virulence genes (*hyl*, *esp*, *cyl*A and *ace*) and antibiotic resistance (*van*A) raises red flags for regulatory approval and probiotic safety. As previously discussed, even if these properties can bring some benefits, safety needs to be a priority in the evaluation of the appropriateness of specific strains to be applied as beneficial microbial cultures. Thus, even if the species has GRAS status, a personalized evaluation for any specific strains regarding his safety needs to be regarded as a priority before any further applications will be suggested.

None of the tested biogenic amine genes were recorded in the DNA from *Ltb. sakei* subsp. *sakei* 2a (Table 1). Moreover, physiological tests performed at 37 °C for the detection of the production of biogenic amines do not generate positive results. Biogenic amines have a significant role in the interaction between probiotic strains and their host. These organic compounds are produced in the processes of decarboxylation of amino acids and are involved in various physiological processes. In general, biogenic amines are considered toxins, and their cumulations are regarded as negative factors for food products and even for probiotics [41]. However, biogenic amines can enhance the growth and survival of probiotic strains because some LAB bacteria can produce biogenic amines as a helpful factor that will assist in adapting to stressful environments, such as acidic conditions in the gastrointestinal tract [42]. Moreover, biogenic amines can act as precursors for the synthesis of other important compounds, contributing to the overall metabolic flexibility and efficiency of the probiotic strains [42].

Biogenic amines can be actively involved in the processes of influence gut health by modulating the gut microbiota, which can act as signaling molecules, affecting the growth and activity of other beneficial microbes in the gut [43]. Moreover, some specific biogenic amines may have immunomodulatory effects, helping to regulate the host’s immune response. This can be beneficial in maintaining a balanced immune system and preventing excessive inflammation [44]. Biogenic amines can be associated with the processes of absorption of certain nutrients by the host. For example, they can increase the bioavailability of amino acids, which are crucial for various bodily functions [43]. However, it is a clear fact that excessive production of biogenic amines can be associated with negative effects on the host, toxicity and can be involved in food spoilage and potential health risks such as histamine intolerance [44]. Therefore, the balance and regulation of biogenic amine production are crucial for both probiotic efficacy and host health.

### 3.2. Safety Evaluation of the Strain

When concerning the safety of LAB, it is important to confirm that strains suggested to be applied as probiotics and other beneficial properties are not hemolytic, gelatinase- or protease-producing, represent resistance/sensitivity to antibiotics according to EFSA recommendations and do not represent excess sensitivity to commonly applied medicaments.

Hemolytic activity is one of the principal safety issues and screening criteria for selecting new putative probiotic strains. Only non-hemolytic (γ-hemolysis) strains can be considered for future investigations and considered safer for human and other animal consumption [45]. Hemolytic activity, particularly β-hemolysis, is directly associated with pathogenicity and destruction of red blood cells, which can lead to consequences regarding health issues [45].

Gelatinase is a type of protease that may improve fermentative properties for some strains in the processes of metabolization of gelatin and some other proteins, helping in their survival and adaptation in various environments and improving specific fermentation processes in the food industry [46]. However, when gelatinase activity is well-expressed in probiotic strains, applied in humans or other animals, gelatinase can also be involved in the degradation processes of the host’s extracellular matrix and mucosal barriers, potentially leading to tissue damage and increased susceptibility to infections [46]. Moreover, proteases, in general, can enable probiotic strains to break down proteins into peptides and amino acids, which are essential for their growth and metabolic activities [47]. Some proteases actively contribute to biofilm formation, which can enhance the resilience and persistence of probiotics in the gastrointestinal tract; however, this may involve negative consequences if some pathogens can be incorporated in multicultural biofilm formations or if the strain with good biofilm formation properties can carry some virulent characteristics [47]. Moreover, proteases produced by probiotics can aid in the digestion of dietary proteins, improving nutrient absorption and overall digestive health [47]. Some specific proteases may have specific immunomodulatory effects, helping to regulate the host’s immune response and maintain gut health [47]. However, they can also be responsible for the proteolysis of the epithelial proteins, contribute to the pathogenicity of some microbial cultures, and even be associated with some types of cancers [48].

While the production of these enzymes by probiotic strains can offer benefits such as enhanced survival and nutrient acquisition, it is essential to balance these advantages with potential risks to the host. Ensuring that probiotic strains are non-hemolytic and that their enzyme production does not harm the host’s tissues is crucial for their safe and effective use.

From 48 tested antibiotics (Table 2), *Ltb. sakei* subsp. *sakei* 2a was resistant only to nalidixic acid (the expected results because this antibiotic is generally effective against Gram-negative bacterial cultures), cotrimazin, ofloxacin, sulfonamide and vancomycin. However, *van*A was already recorded in the DNA from *Ltb. sakei* subsp. *sakei* 2a (Table 1). However, none of the recommended antibiotics by EFSA regarded the safety of *Ltb. sakei* strains were showing safety concerns.

Growth of *Ltb. sakei* subsp. *sakei* 2a was not inhibited by commercial drugs from different generic groups. The inhibitory effect on the growth of *Ltb. sakei* subsp. *sakei* 2a was recorded only in the presence of Arotin [selective serotonin reuptake inhibitor antidepressant] MIC 1.0 mg/mL, Atlansil [Antiarrhythmic] MIC 0.625 mg/mL, Diclofenac potassium [non-steroidal anti-inflammatory drug (NSAID)] MIC 2.5 mg/mL and Spidufen [NSAID] MC 15.0 mg/mL. Only two antibiotics tested in this study, Amoxil and Urotrobel, inhibited the growth of *Ltb. sakei* subsp. *sakei* 2a with a MIC of <0.5 mg/mL and 5.0 mg/mL, respectively (Table 3).

The interaction between commercial drugs and probiotic strains is a relevant topic, as it can influence the efficacy and safety of both the medications and the probiotics. The interactions between antibiotics and probiotics are already a well-discussed topic and have been raised regarding how antibiotics and probiotics need to be received by patients. It is a clear fact that antibiotics can significantly reduce the population of probiotic bacteria in the gut. This is because antibiotics often do not distinguish between pathogenic and beneficial bacteria associated with their nonspecific mode of action. On the other side, probiotics can help mitigate some of the adverse effects of antibiotics, such as antibiotic-associated diarrhea, by restoring the balance of the gut microbiota [49].

Some probiotics can affect the absorption of certain medications. For example, they might alter the gut environment or interact with the drug directly, potentially affecting its bioavailability. Some probiotics can modulate the immune system, which might interfere with the action of immunosuppressive drugs. This interaction could potentially reduce the effectiveness of immunosuppressants [50]. Moreover, some probiotics may help alleviate some gastrointestinal side effects of chemotherapy, such as mucositis and diarrhea, by maintaining a healthy gut microbiota [49] or improve reducing the side effects [51].

The cumulation of some non-antibiotics commonly used drugs may cumulate in the gut environment, and if they present inhibitory effects versus probiotic strains, this may influence the viability of the probiotics. It has already been reported that some NSAID drugs have these inhibitory effects [52]. Moreover, the concern is the drugs that are associated with long-term treatments because some of them can cumulate and as well further present some inhibitory properties versus some probiotic strains and gut microbiota in general [53]. This is a topic that needs more attention and further studies with the aim of optimizing the application of drugs and probiotics and taki advantage of both in the name of better health for patients.

### 3.3. Growth at Different pH and Bile Concentrations

**Figure 1:**
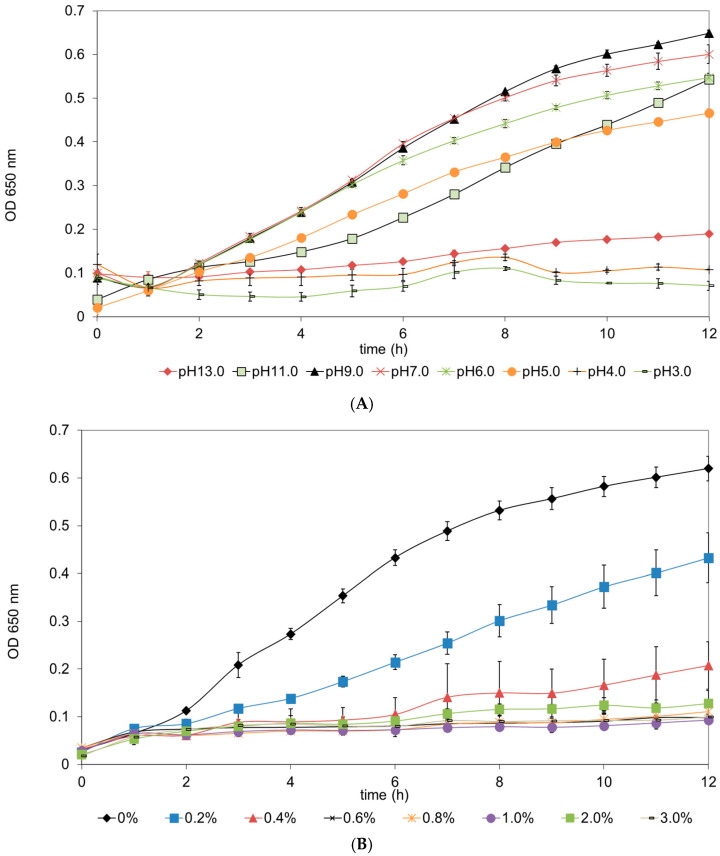
(**A**) Growth of *Latilactobacillus sakei* subsp. *sakei* 2a in MRS broth (Difco) at different pH levels; (**B**) supplemented with different concentrations of oxbile.

*Ltb. sakei* subsp. *sakei* 2a showed good microbial growth when cultured in MRS broth (Difco) adjusted to pH 5.0, 6.0, 7.0 and 9.0 and MRS broth containing 0.2% and 0.4% (*w*/*v*) oxbile (Figure 1). These results are expected, taking into consideration the identity of the studied strain [54]. Similar results were reported for other lactobacilli [26]. However, when the same experimental procedures were performed for representatives from the genus Enterococcus, a better adaptation of enterococci compared with lactobacilli to extreme levels of pH (acid or basic) or the presence of oxbile was observed [26].

Survival in different pH environments and the presence of oxbile are critical factors for the efficacy of probiotic strains and their benefits to the host. The probiotics will initially need to survive passage via the stomach, where pH can reach a pH of 1.5–2.5 [55], and further, when reaching the gut environments, depending on the physiological conditions presence of oxbile can reach even from 1 to 10 mM after a meal [56]. Moreover, oxbile acids are important and contribute to the digestion and absorption of fats and fat-soluble vitamins [57]. Probiotics need to tolerate these oxbile acid levels [58]. Moreover, exposure to sub-lethal acidic conditions can lead to the enhancement of tolerance to other environmental stress conditions and, as a consequence, improve their survival in the gut environment [59].

Probiotic cultures with acid tolerance specificity will effectively survive the acidic stomach environment and passage and have better chances to further colonize the intestines and contribute to a balanced gut microbiota, perform their benefits and improve digestive health [55].

The gut environment is a complex part of the digestive system. The presence of oxbile is produced, and their concentrations can reach up to 10 mM, and they play essential contributions in processes associated with the digestion of lipid-rich food products [56]. However, oxbile acid can show negative effects on lactobacilli [60] and is directly associated with their viability and survival [26]. Thus, oxbile tolerance is a key criterion for selecting effective probiotic strains [61] and simplified gastrointestinal models can serve as effective screening tools for the preselection of putative probiotics for further research investigations. Moreover, some probiotics can deconjugate bile acids through bile salt hydrolase (BSH) activity, which can influence their metabolic activities and survival [62]. Probiotics strains with BSH activity can contribute to the deconjugation of bile salts and, as a consequence, can contribute to the reduction of cholesterol levels by preventing its reabsorption in the intestines [61]. Probiotic cultures with oxbile tolerance will have better colonization viability in the small intestine and may contribute further to healthier gut microbiota and improved digestion [61].

The ability of *Ltb. sakei* subsp. *sakei* 2a to survive in different pH levels and in the presence of oxbile is crucial for its further effectiveness as a beneficial probiotic strain. These survival properties can ensure that probiotics can reach their target sites in the gastrointestinal tract, where they can exert their beneficial effects on the host’s health.

### 3.4. Auto-Aggregation and Co-Aggregation

**Figure 2:**
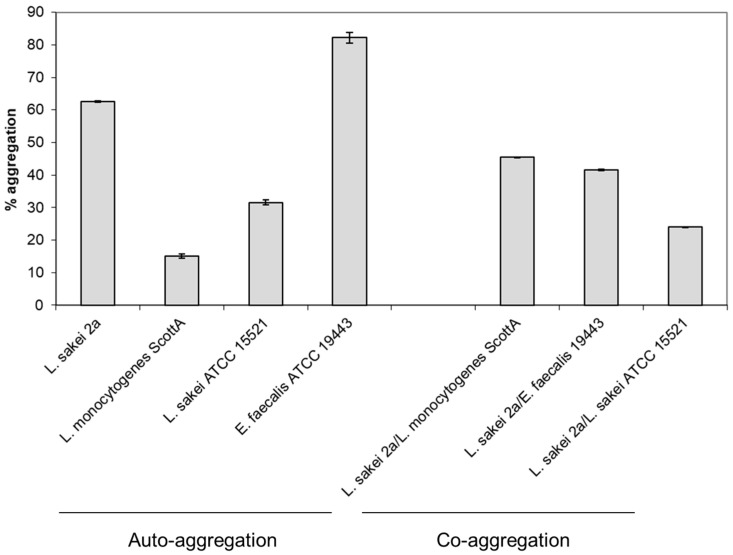
Auto-aggregation and co-aggregation of *Latilactobacillus sakei* subsp. *sakei* 2a. *Enterococcus faecium* ATCC19443, *Lactobacillus sakei* ATCC15521 and *Listeria monocytogenes* ScottA.

In the evaluation of the aggregation (auto- and co-aggregation) properties of *Ltb. sakei* subsp. *sakei* 2a different values were recorded: auto-aggregation of *Ltb*. *sakei* 2a was 62.59% (Figure 2). Different levels of co-aggregation were recorded between *Ltb. sakei* subsp. *sakei* 2a and *E. faecalis* ATCC19443, *Ltb. sakei* ATCC15521 and *L. monocytogenes* ScottA (Figure 2). Aggregation, including both auto-aggregation and co-aggregation, is a crucial property for probiotic strains and has significant implications for the host.

Auto-aggregation, a process where probiotic cells adhere to each other, plays an essential role in the ability of probiotics to colonize the gastrointestinal tract. The auto-aggregation (called, in some cases, self-adherence) contributes to biofilm formation processes and, as a consequence, contributes to the protection of the bacterial cultures from environmental stresses and enhances their persistence in the gut [63]. Moreover, it was suggested that by the formation of aggregates and biofilm, probiotic cells can better resist adverse conditions such as low pH and oxbile salts, and this can significantly improve their survival rates during gastrointestinal transit [63]. Effective colonization by probiotics with high auto-aggregating properties can result in a more stable and resilient gut microbiota, where probiotics can perform their benefits, including maintaining gut health and preventing infections [27]. Moreover, when a biofilm by probiotics strains is formed through auto-aggregation, this can improve the gut barrier function, reducing the permeability to pathogens and even some toxins [27].

On the other side, co-aggregation refers to the ability of microbial cultures (including probiotics) to adhere to others from different strain cultures (including pathogenic bacterial representatives). As a consequence, this interaction can improve processes of inhibition of pathogens by preventing them from attaching to the gut epithelium [63]. Moreover, if the beneficial strain is producing antimicrobial metabolites (including bacteriocins) versus the co-aggregation partner, the co-aggregation properties will improve possibilities for the inhibition of the mentioned pathogens by the produced bacteriocins (or other antimicrobial metabolites) because close contacts will be established between co-aggregation partners [64]. As a consequence, via co-aggregating with pathogens, probiotics can competitively exclude these harmful bacteria from the gut environment, thereby reducing the risk of infections [63]. Moreover, it was suggested that the interaction between probiotics and pathogens through co-aggregation can modulate the host’s immune response, enhancing the body’s ability to fight off infections [65]. The aggregation properties of probiotics, both auto-aggregation and co-aggregation, are essential for their effectiveness in promoting gut health and protecting against pathogens. These properties enhance the colonization and persistence of probiotics in the gut, contribute to the stability of the gut microbiota, and provide a protective barrier against infections.

### 3.5. Role of Prebiotics on the Bacterial Growth and Production of Bacteriocins

For the evaluation of the effect of FOS on bacterial growth and production of bacteriocin by *Ltb. sakei* subsp. *sakei* 2a, a modified MRS broth was prepared, where glucose was replaced with the same level (20 g/L) FOS. It was well-observed that glucose was clearly supporting bacterial growth (Table 4). However, the replacement of glucose by FOS was not providing optimal growth for *Ltb. sakei* subsp. *sakei* 2a. At 24 h from bacterial growth, the OD600 nm for culture grown in MRS/glucose was corresponding to 2.062 (calculated after appropriate dilutions), while when MRS/FOS was used for growing conditions, only 0.62 OD was recorded (Table 4). It was expected that in reduced bacterial growth, bacteriocin production would not be the same. In the experiment with MRS/glucose, the recorded activity against *L. monocytogenes* ScottA was 3200 AU/mL, and when MRS/FOS was used, activity was only 200 AU/mL. However, taking into consideration bacterial population density (and if we can stimulate approximative CFU/mL), then can be observed that estimated AU/CFU are reasonably similar. Rwubuzizi et al. [66] suggested that for the correct comparison of bacteriocin production, we need to take into consideration the level of bacterial growth and not only compare the AU/mL.

Prebiotic properties for FOS were suggested and already explored, showing its role in promoting bacterial growth and the production of antimicrobial peptides, which are crucial for maintaining gut health and preventing infections. However, not all bacterial cultures have appropriate enzymatic systems to hydrolyze the FOS to simple sugars that can be further metabolized and used as energy sources by bacterial cultures. Bifidobacteria have a remarkable ability to metabolize FOS, which is a key factor in their role as beneficial gut microbes. Bifidobacteria produce β-fructosidases (inulinases) that hydrolyze FOS into simpler sugars that can be utilized for growth and energy [67]. However, based on observed results (Table 4), *Ltb. sakei* subsp. *sakei* 2a cannot be considered as a producer of β-fructosidases, observed based on very limited bacterial growth in our experiment. Moreover, not all species of Bifidobacterium are equal producers of β-fructosidases. For instance, Bifidobacterium adolescentis has been shown to grow well and produce significant amounts of organic acids when metabolizing both short- and long-chain FOS [68].

## 4. Conclusions

Besides all the beneficial properties studied for various LAB, special attention needs to be paid to the possible presence of virulence factors, production of biogenic amines and antibiotic resistance. These virulence determinants have been well-detected and studied in enterococci and streptococci; however, in the last few years, reports on the presence of virulence factors in otherwise GRAS lactobacilli have been showing potential upcoming problems. Horizontal gene transfer of virulence factors between pathogenic and LAB, including probiotics, is a highly possible scenario in case of uncontrolled application of probiotics. FOS are essential for enhancing the growth of beneficial gut bacteria and stimulating the production of antimicrobial peptides; however, the targeted microbial culture needs to be positive for β-fructosidases, and this will allow them to primary hydrolyze the FOS to the simple carbohydrates and further used by the microbial cultures in their metabolite processes. These effects contribute to a healthier gut environment, improved immune function, and protection against pathogenic infections.

## Figures and Tables

**Table 1:** Presence of genes in DNA extracted from *Latilactobacillus sakei* subsp. *sakei* 2a. Sequence of applied primers and reference works for the PCR conditions were indicated.

	Detection of the Target Gen in the DNA from *Ltb. sakei* subsp. *sakei* 2a	Primers (5′–3′)	Reference
Virulence Genes			
*gel*E (gelatinase)	−	TAT GAC AAT GCT TTT TGG GAT AGA TGC ACC CGA AAT AAT ATA	[12]
*hyl* (hyaluronidase)	+	ACA GAA GAG CTG CAG GAA ATG GAC TGA CGT CCA AGT TTC CAA	[12]
*asa*1 (aggregation substance)	−	GCA CGC TAT TAC GAA CTA TGA TAA GAA AGA ACA TCA CCA CGA	[12]
*esp* (enterococcal surface protein)	+	AGA TTT CAT CTT TGA TTC TTG AAT TGA TTC TTT AGC ATC TGG	[12]
*cyl*A (cytolisin)	+	ACT CGG GGA TTG ATA GGC GCT GCT AAA GCT GCG CTT	[12]
*efa*A (endocarditis antigen)	−	GCC AAT TGG GAC AGA CCC TC CGC CTT CTG TTC CTT CTT TGG C	[13]
*ace* (adhesion of collagen)	+	GAA TTG AGC AAA AGT TCA ATC G GTC TGT CTT TTC ACT TGT TTC	[13]
Vancomycin resistance			
*van*A	+	TCT GCA ATA GAG ATA GCC GC GGA GTA GCT ATC CCA GCA TT	[13]
*van*B	−	GCT CCG CAG CCT GCA TGG ACA ACG ATG CCG CCA TCC TCC TGC	[13]
*van*C	−	ATC CAA GCT ATT GAC CCG CT TGT GGC AGG ATC GTT TTC AT	[14]
*van*D	−	TGT GGG ATG CGA TAT TCA A TGC AGC CAA GTA TCC GGT AA-3	[14]
*van*E	−	TGT GGT ATC GGA GCT GCA G GTC GAT TCT CGC TAA TCC	[14]
*van*G	−	GAA GAT GGT ACT TTG CAG GGC A AGC CGC TTC TTG T A T CCG TTT T	[14]
Biogenic amines			
*hdc*1 (histidine decarboxylase)	−	AGA TGG TAT TGT TTC TTA TG AGA CCA TAC ACC ATA ACC TT	[15]
*hdc*2 (histidine decarboxylase)	−	AAY TCN TTY GAY TTY GAR AAR GAR G ATN GGN GAN CCD ATC ATY TTR TGN CC	[15]
*tdc* (tyrosine decarboxylase)		GAY ATN ATN GGN ATN GGN YTN GAY CAR G CCR TAR TCN GGN ATA GCR AAR TCN GTR TG	[15]
*odc* (ornithine decarboxylase)	−	GTN TTY AAY GCN GAY AAR CAN TAY TTY GT ATN GAR TTN AGT TCR CAY TTY TCN GG	[15]
Beneficial			
*map*	+	TGG ATT CTG CTT GAG GTA AG GAC TAG TAA TAA CGC GAC CG	[16]
*mub*	+	GTA GTT ACT CAG TGA CGA TCA ATG TAA TTG TAA AGG TAT AAT CGG AGG	[16]
*eftu*	+	TTC TGG TCG TAT CGA TCG TG CCA CGT AAT AAC GCA CCA AC	[16]
*core* (GABA)	+	CCT CGA GAA GCC GATC GCT TAG TTC G TCA TAT TGA CCG GTA TAA GTG ATG CCC	[16]

**Table 2:** The antibiotic resistance of *Latilactobacillus sakei* subsp. *sakei* 2a was assessed using antibiotic discs (Cefar Diagnóstica Ltd.a., São Paulo, Brazil) on MRS agar plates. The inhibition zones were measured using a ruler.

Antibiotics	Inhibition Zone		
	Average, mm	SD	% Error
Ampicillin + Suebactam	20.00	1.41	7.07
Bacitracin	24.00	0.00	0.00
Ampicillin + Suebactam	25.50	0.71	2.77
Amoxicillin + clavulonic acid	21.50	0.71	3.29
Nalidixic acid	0.0	0.0	0.0
Amikacin	15.00	1.41	9.43
Cefaclor	21.50	0.71	3.29
Cafalotin	20.00	0.00	0.00
Cafazolin	19.50	2.12	10.88
Cefepime	14.50	0.71	4.88
Cefotaxime	17.00	0.00	0.00
Cefoxitin	22.00	1.41	6.43
Ceftazidime	12.50	0.71	5.66
Ceftiofur	22.00	2.83	12.86
Ceftriaxone	15.50	0.71	4.56
Cefuroxime	19.00	0.00	0.00
Ciprofloxacin	11.50	0.71	6.15
Claritromycin	23.00	0.00	0.00
Clindamycin	31.50	2.12	6.73
Cloramphenicol	25.00	0.00	0.00
Cotrimazole	0.0	0.0	0.0
Doxycycline	24.50	0.71	2.89
Enrofloxacin	11.00	0.00	0.00
Erythromycin	22.50	0.71	3.14
Spectinomycin	23.50	2.12	9.03
Streptomycin	23.00	1.41	6.15
Florfenicol	25.00	0.00	0.00
Gentamicin	15.50	0.71	4.56
Kanamycin	12.00	0.00	0.00
Linezolid	27.00	2.83	10.48
Identifar	12.00	2.83	23.57
Imipenem	27.00	1.41	5.24
Minocycline	30.00	0.00	0.00
Moxifloxacin	14.50	0.71	4.88
Neomycin	15.25	0.35	2.32
Nitrofurantoin	21.50	0.71	3.29
Penicillin G	24.50	0.71	2.89
Piperacillina + Tazobactam	20.00	0.00	0.00
Ofloxacin	0.0	0.0	0.0
Oxacillin	21.00	1.41	6.73
Rifampicin	27.50	2.12	7.71
Sulfonamide	0.0	0.0	0.0
Teicoplanin	15.00	0.00	0.00
Tobramycin	14.50	0.71	4.88
Tetracycline	24.50	0.71	2.89
Tilmicosin	12.00	0.00	0.00
Timethroprim	27.50	3.54	12.86
Vancomycin	0.0	0.0	0.0

**Table 3:** Effect of commercial drugs on growth of *Latilactobacillus sakei* subsp. *sakei* 2a. Commercial names, active substances and medical groups are indicated. MIC was estimated for the drugs with inhibitory effect against *Latilactobacillus sakei* subsp. *sakei* 2a.

Commercial Name	Concentration (mg/mL)	Active Substance	Medicament Class	*Ltb. sakei* subsp. *sakei* 2a
				Inhibition Zone (mm) [MIC (mg/mL)]
AAS	20	Acetylsalicylic acid	Analgesic/Antipyretic	0
Amoxil	100	Amoxicillin	β-Lactam antibiotic (Penicilin)	42 [<0.4]
Antak	30	Ranitidine hydrochloride	Histamine H2-receptor antagonist that inhibits stomach acid production (Proton pump inhibitor)	0
Arotin	4	Paroxetine	selective serotonin reuptake inhibitor (SSRI) antidepressant	15 [1.0] 0
Aspirina	100	Acetylsalicylic acid	Analgesic/Antipyretic	
Atlansil	40	Amiodarone	Antiarrhythmic	10 [0.625]
Cataflam	10	Diclofenac potassium	Non-steroidal anti-inflammatory drug (NSAID)	15 [2.5]
Celebra	40	Celecoxib	NSAID	0
Clorana	5	Hydrochlorothiazide	Diuretic	0
Coristina R		Acetylsalicylic acid. Pheniramine maleate. Phenylephrine hydrochloride. Cafein	Analgesic/Antipyretic/Antihistaminic/Decongestant	0
Diclofenac potassico *	10	Diclofenac potassium	NSAID	16 [2.5]
Diclofenaco potassico *	10	Diclofenac potassium	NSAID	14 [2.5]
Dorflex	10	Orphenadrine citrate. Metamizole sodium. Cafein	Analgesic	0
Doxuran	0.8	Doxazosin	Antihypertensive/treatment of prostatic hyperplasia	0
Dramin	20	Dimenhydrinate	Antiemetic	0
Fenergan	5	Promethazine hydrochloride	Antihistaminic	0
Fluimucil	8	Acetylcysteine	Mucolitic agent	0
Flutec	30	Fluconazole	Antifungal	0
Higroton	10	Chlorthalidone	Thiazide diuretic	0
Medley	4	Omeprazole	Proton pump inhibitor	0
Neosaldina	60	Metamizole sodium. Isometheptene mucate. Cafein	Analgesic	0
Nimesulida	20	Nimesulide	NSAID	0
Nisulid	20	Nimesulide	NSAID	0
Redulip	3	Sibutramine hydrochloride monohydrate	Anorexiant/Sympathomimetic	0
Seki	3.54	Cloperastine	Antitussives (central and periferic mode of action)	0
Spidufen	120	Ibuprofen arginine	NSAID	22 [15.0]
Superhist	80	Acetylsalicylic acid Pheniramine maleate Phenylephrine hydrochloride	Analgesic/Antipyretic/Antihistaminic/Decongestant	0
Tylenol	150	Paracetamol	Analgesic/Antipyretic	0
Tylex	6	Paracetamol. Codein	Analgesic	0
Urotrobel	80	Norfloxacin	Antibiotic	8 [10.0]
Yasmin	0.6	Ethinylestradiol. Drospirenone	Contraceptive	0
Zestril	4	Lisinopril	Antihypertensive (Angiotensin-converting enzyme (ACE) inhibitor)	0
Zocor	2	Simvastatin	Hypolipidemic	0
Zyrtec	2	Cetirizine hydrochloride	Antihistaminic	0

* Produced by two different companies.

**Table 4:** Bacterial growth, changes in pH and production of bacteriocin by *Latilactobacillus sakei* subsp. *sakei* 2a cultured in MRS/glucose and MRS/FOS (at 20 g/L). Bacteriocin activity was evaluated against *Listeria monocytogenes* ScottA.

	MRS/Glucose			MRS/FOS		
Time (h)	pH	OD	AU/mL	pH	OD	AU/mL
0	6.51	0.018	0	6.49	0.012	0
8	6.10	0.23	200	6.05	0.092	0
12	5.08	1.714	800	5.89	0.40	200
24	3.92	2.062	3200	5.67	0.62	200

## Data Availability

The data presented in this study are available on request from the corresponding author. The data are not publicly available due to privacy restrictions.

## References

[1] Kim S.S., Kim S. (2022). Impact and prospect of the fourth industrial revolution in food safety: Mini-review. Food Sci. Biotechnol..

[2] Reuben R.C., Torres C. (2024). Bacteriocins: Potentials and prospects in health and agrifood systems. Arch. Microbiol..

[3] Choi G.H., Holzapfel W.H., Todorov S.D. (2023). Diversity of the bacteriocins, their classification and potential applications in combat of antibiotic resistant and clinically relevant pathogens. Crit. Rev. Microbiol..

[4] Younes M., Aggett P., Aguilar F., Crebelli R., Dusemund B., Filipič M., Frutos M.J., Galtier P., Gundert-Remy U., EFSA Panel on Food Additives and Nutrient Sources added to Food (ANS) (2017). Safety of nisin (E 234) as a food additive in the light of new toxicological data and the proposed extension of use. EFSA J..

[5] Hill C., Guarner F., Reid G., Gibson G.R., Merenstein D.J., Pot B., Morelli L., Canani R.B., Flint H.J., Salminen S. (2014). The International Scientific Association for Probiotics and Prebiotics consensus statement on the scope and appropriate use of the term probiotic. Nat. Rev. Gastroenterol. Hepatol..

[6] Todorov S.D., Lima J.M.S., Bucheli J.E.V., Popov I.V., Tiwari S.K., Chikindas M.L. (2024). Probiotics for aquaculture: Hope, truth, and reality. Probiotics Antimicrob. Proteins.

[7] Kumar A., Green K.M., Rawat M. (2024). A comprehensive overview of postbiotics with a special focus on discovery techniques and clinical applications. Foods.

[8] Liserre A.L., Landgraf M., Destro M.T., Franco B.D.G.M. (2002). Inhibition of *Listeria monocytogenes* by a bacteriocinogenic *Lactobacillus sake* strain in modified atmosphere-packaged Brazilian sausage. Meat Sci..

[9] Malheiros P.S., Sant’Anna V., Todorov S.D., Franco B.D. (2015). Optimization of growth and bacteriocin production by *Lactobacillus sakei* subsp. *sakei* 2a. Braz. J. Microbiol..

[10] Martinez R.C.R., Staliano C.D., Vieira A.D.S., Villarreal M.L.M., Todorov S.D., Saad S.M.S., Franco B.D.G.M. (2015). Bacteriocin production and inhibition of *Listeria monocytogenes* by *Lactobacillus sakei* 2a in potentially synbiotic cheese spread. Food Microbiol..

[11] Todorov S.D., Penna A.L.B., Venema K., Holzapfel W.H., Chikindas M.L. (2023). Recommendations for the use of standardized abbreviations for the former Lactobacillus genera, reclassified in the year 2020 (Opinion). Benef. Microbes.

[12] Vankerckhoven V., Van Autgaerden T., Vael C., Lammens C., Chapelle S., Rossi R., Jabes D., Goossens H. (2024). Development of a multiplex PCR for the detection of *asa*1, *gel*E, *cyl*A, *esp*, and *hyl* genes in enterococci and survey for virulence determinants among European hospital isolates of *Enterococcus faecium*. J. Clin. Microbiol..

[13] Martin-Platero A.M., Valdivia E., Maqueda M., Martinez-Bueno M. (2009). Characterization and safety evaluation of enterococci isolated from Spanish goats’ milk cheeses. Int. J. Food Microbiol..

[14] Lima J.M.S., Carneiro K.O., Pinto U.M., Todorov S.D. (2024). Bacteriocinogenic anti-listerial properties and safety assessment of *Enterococcus faecium* and *Lactococcus garvieae* strains isolated from Brazilian artisanal cheesemaking environment. J. Appl. Microbiol..

[15] Rivas P., Alonso J., Moya J., de Gorgolas M., Martinell J., Guerrero M.L.F. (2005). The impact of hospital-acquired infections on the microbial etiology and prognosis of late-onset prosthetic valve endocarditis. Chest.

[16] Valledor S.J.D., Dioso C.M., Vazquez Bucheli J.E., Park Y.J., Suh D.H., Jung E.S., Kim B., Holzapfel W.H., Todorov S.D. (2022). Characterization and safety evaluation of two beneficial, enterocin-producing *Enterococcus faecium* strains isolated from kimchi, a Korean fermented cabbage. Food Microbiol..

[17] Santini V., Fenaux P., Mufti G.J., Hellström-Lindberg E., Silverman L.R., List A., Gore S.D., Seymour J.F., Backstrom J., Beach C.L. (2010). Management and supportive care measures for adverse events in patients with myelodysplastic syndromes treated with azacitidine. Eur. J. Haematol..

[18] dos Santos K.M.O., de Matos C.R., Salles H.O., Franco B.D.G.M., Arelano K., Holzapfel W.H., Todorov S.D. (2020). Exploring beneficial/virulence properties of two dairy-related strains of *Streptococcus infantarius* subsp. *infantarius*. Probiotics Antimicrob. Proteins.

[19] Domingos-Lopes M.F.P., Stanton C., Ross P.R., Dapkevicius M.L.E., Silva C.C.G. (2017). Genetic diversity, safety and technological characterization of lactic acid bacteria isolated from artisanal Pico cheese. Food Microbiol..

[20] Fugaban J.I.I., Vazquez Bucheli J.E., Holzapfel W.H., Todorov S.D. (2021). Characterization of partially purified bacteriocins produced by *Enterococcus faecium* strains isolated from soybean paste active against *Listeria* spp. and vancomycin-resistant *Enterococci*. Microorganisms.

[21] Bover-Cid S., Holzapfel W.H. (1999). Improved screening procedure for biogenic amine production by lactic acid bacteria. Int. J. Food Microbiol..

[22] Gajic I., Kabic J., Kekic D., Jovicevic M., Milenkovic M., Mitic Culafic D., Trudic A., Ranin L., Opavski N. (2022). Antimicrobial susceptibility testing: A comprehensive review of currently used methods. Antibiotics.

[23] Furtado D.N., Todorov S.D., Landgraf M., Destro M.T., Franco B.D.G.M. (2014). Bacteriocinogenic *Lactococcus lactis* subsp. *lactis* DF04Mi isolated from goat milk: Evaluation of the probiotic potential. Braz. J. Microbiol..

[24] Vera Pingitore E., Todorov S.D., Sesma F., Franco B.D. (2012). Application of bacteriocinogenic Enterococcus mundtii CRL35 and Enterococcus faecium ST88Ch in the control of *Listeria monocytogenes* in fresh Minas cheese. Food Microbiol..

[25] Ramiah K., van Reenen C.A., Dicks L.M. (2008). Surface-bound proteins of *Lactobacillus plantarum* 423 that contribute to adhesion of Caco-2 cells and their role in competitive exclusion and displacement of *Clostridium sporogenes* and *Enterococcus faecalis*. Res. Microbiol..

[26] Todorov S.D., Dicks L.M.T. (2008). Evaluation of lactic acid bacteria from kefir, molasses and olive brine as possible probiotics based on physiological properties. Ann. Microbiol..

[27] Monteagudo-Mera A., Rastall R.A., Gibson G.R., Charalampopoulos D., Chatzifragkou A. (2019). Adhesion mechanisms mediated by probiotics and prebiotics and their potential impact on human health. Appl. Microbiol. Biotechnol..

[28] Hynönen U., Kant R., Lähteinen T., Pietila T.P., Beganovic J., Smidt H., Uroic K., Avall-Jaaskelainen S., Palva A. (2014). Functional characterization of probiotic surface layer protein-carrying *Lactobacillus amylovorus* strains. BMC Microbiol..

[29] Sabna B.S., Thankappan B., Mahendran R., Muthusamy G., Selta D.R.F., Angayarkanni J. (2021). Evaluation of GABA production and probiotic activities of *Enterococcus faecium* BS5. Probiotics Antimicrob. Proteins.

[30] Tette F.-M., Kwofie S.K., Wilson M.D. (2022). Therapeutic anti-depressant potential of microbial GABA produced by *Lactobacillus rhamnosus* strains for GABAergic signaling restoration and inhibition of addiction-induced HPA axis hyperactivity. Curr. Issues Mol. Biol..

[31] Kaur S., Sharma P., Mayer M.J., Neuert S., Narbad A., Kaur S. (2023). Beneficial effects of GABA-producing potential probiotic *Limosilactobacillus fermentum* L18 of human origin on intestinal permeability and human gut microbiota. Microb. Cell Factories.

[32] Suvorov A. (2020). What is wrong with enterococcal probiotics?. Probiotics Antimicrob. Proteins.

[33] EFSA Panel on Additives and Products or Substances used in Animal Feed (FEEDAP) (2012). Scientific Opinion on Title of the Opinion, EFSA Journal. www.efsa.europa.eu/efsajournal.

[34] Anisimova E.A., Yarullina D.R. (2019). Antibiotic resistance of *Lactobacillus* Strains. Curr. Microbiol..

[35] Zheng M., Zhang R., Tian X., Zhou X., Pan X., Wong A. (2017). Assessing the risk of probiotic dietary supplements in the context of antibiotic resistance. Front. Microbiol..

[36] Ahmed M.O., Baptiste K.E. (2018). Vancomycin-resistant *Enterococci*: A review of antimicrobial resistance mechanisms and perspectives of human and animal health. Microb. Drug Resist..

[37] Gueimonde M., Sanchez B., los Reyes-Gavilan C.G., Morgolles A. (2013). Antibiotic resistance in probiotic bacteria. Front. Microbiol..

[38] Elshaghabee F.M.F., Rokana N. (2022). Mitigation of antibiotic resistance using probiotics, prebiotics and synbiotics. A review. Environ. Chem. Lett..

[39] Kiruthiga A., Padmavathy K., Shabana P., Neveenkumar V., Gnanadeskan S., Malaiyan J. (2020). Improved detection of *esp*, *hyl*, *asa*1, *gel*E, *cyl*A virulence genes among clinical isolates of Enterococci. BMC Res. Notes.

[40] Huang K., Shi W., Yang B., Wang J. (2023). The probiotic and immunomodulation effects of *Limosilactobacillus reuteri* RGW1 isolated from calf feces. Front. Cell. Infect. Microbiol..

[41] Wójcik W., Łukasiewicz M., Puppel K. (2021). Biogenic amines: Formation, action and toxicity—A review. J. Sci. Food Agric..

[42] Shanenko E.F., Nikolaev Y.A., Ganina V.I., Serykh I.N., Oleskin A.V., Mukhamedzanova T.G., Grigorieva N.V., El-Registan G.I. (2022). Synthesis of biogenic amines by lactic acid bacteria on media of plant and animal origin. Microbiology.

[43] Neis E.P.J.G., Dejong C.H.C., Rensen S.S. (2015). The role of microbial amino acid metabolism in host metabolism. Nutrients.

[44] Lee N.K., Kim W.S., Paik H.D. (2019). *Bacillus* strains as human probiotics: Characterization, safety, microbiome, and probiotic carrier. Food Sci. Biotechnol..

[45] Tran K.D., Le-Thi L., Vo H.H., Dinh-Thi T.-V., Nguyen-Thi T., Phan N.-H., Nguyen K.-U. (2024). Probiotic properties and safety evaluation in the invertebrate model host *Galleria mellonella* of the *Pichia kudriavzevii* YGM091 strain isolated from fermented goat milk. Probiotics Antimicrob. Proteins.

[46] Chaudhari A., Pandey S., Dwivedi M.K., Dwivedi M.K., Amaresan N., Sankaranarayanan A., Begum R. (2022). Determination of gelatinases, glycosidases, and enolase production. Biosafety Assessment of Probiotic Potential. Methods and Protocols in Food Science.

[47] da Silva L.A., Lopes Neto J.H.P., Cardarelli H.R. (2019). Safety and probiotic functionality of isolated goat milk lactic acid bacteria. Ann. Microbiol..

[48] Mitschke J., Burk U.C., Reinheckel T. (2019). The role of proteases in epithelial-to-mesenchymal cell transitions in cancer. Cancer Metastasis Rev..

[49] Bradford G., Asgari B., Smit B., Hatje E., Kuballa A., Katouli M. (2024). The efficacy of selected probiotic strains and their combination to inhibit the interaction of adherent-invasive *Escherichia coli* (AIEC) with a co-culture of Caco-2:HT29-MTX cells. Microorganisms.

[50] Raval S.D., Archana G. (2024). Evaluation of synbiotic combinations of commercial probiotic strains with different prebiotics in *in vitro* and *ex vivo* human gut microcosm model. Arch. Microbiol..

[51] López-Gómez L., Alcorta A., Abalo R. (2023). Probiotics and probiotic-like agents against chemotherapy-induced intestinal mucositis: A narrative review. J. Pers. Med..

[52] Maseda D., Ricciotti E. (2020). NSAID-gut microbiota interactions. Front. Pharmacol..

[53] Todorov S.D., Botes M., Danova S.T., Dicks L.M.T. (2007). Probiotic properties of *Lactococcus lactis* subsp. *lactis* HV219, isolated from human vaginal secretions. J. Appl. Microbiol..

[54] de Vos P., Garrity G.M., Jones D., Kreig N.R., Ludwig W., Rainey F.A., Schleifer K.-H., Whitman W.B. (2009). The Firmicutes. Bergey’s Manual of Systematic Bacteriology.

[55] Fujimori S. (2020). Gastric acid level of humans must decrease in the future. World J. Gastroenterol..

[56] Begley M., Gahan Cormac G.M., Hill C. (2005). The interaction between bacteria and bile. FEMS Microbiol. Rev..

[57] Guzior D.V., Quinn R.A. (2021). Review: Microbial transformations of human bile acids. Microbiome.

[58] Sun Y., Wang X., Li L., Zhong C., Zhang Y., Yang X., Li M., Yang C. (2024). The role of gut microbiota in intestinal disease: From an oxidative stress perspective. Front. Microbiol..

[59] Kulkarni S., Haq S.F., Samant S., Sukumaran S. (2018). Adaptation of *Lactobacillus acidophilus* to thermal stress yields a thermotolerant variant which also exhibits improved survival at pH 2. Probiotics Antimicrob. Proteins.

[60] Bernard J.N., Chinnaiyan V., Almeda J., Catala-Valentin A., Andl C.D. (2023). *Lactobacillus* sp. facilitate the repair of DNA damage caused by bile-induced reactive oxygen species in experimental models of gastroesophageal reflux disease. Antioxidants.

[61] da Silva T.F., Glória R., Americo M.F., Freitas A.S., de Jesus L.C.L., Barroso F.A.L., Laguna J.G., Coelho-Rocha N.D., Tavares L.M., le Loir Y. (2024). Unlocking the potential of probiotics: A comprehensive review on research, production, and regulation of probiotics. Probiotics Antimicrob. Proteins.

[62] Horackova S., Vesela K., Klojdova I., Bercikova M., Plackova M. (2020). Bile salt hydrolase activity, growth characteristics and surface properties in *Lactobacillus acidophilus*. Eur. Food Res. Technol..

[63] Collado M.C., Meriluoto J., Salminen S. (2008). Adhesion and aggregation properties of probiotic and pathogen strains. Eur. Food Res. Technol..

[64] Todorov S.D., Furtado D.N., Saad S.M.I., Tome E., Franco B.D.G.M. (2011). Potential beneficial properties of bacteriocin-producing lactic acid bacteria isolated from smoked salmon. J. Appl. Microbiol..

[65] Zawistowska-Rojek A., Kośmider A., Stępień K., Tyski S. (2022). Adhesion and aggregation properties of *Lactobacillaceae* strains as protection ways against enteropathogenic bacteria. Arch. Microbiol..

[66] Rwubuzizi R., Fugaban J.I.I., Holzapfel W.H., Todorov S.D. (2024). Media optimization for bacteriocin production by *Enterococcus faecium* strains isolated from traditional Korean soybean paste. Acta Microbiol. Bul..

[67] McKellar R.C., Modler H.W. (1989). Metabolism of fructo-oligosaccharides by *Bifidobacterium* spp.. Appl. Microbiol. Biotechnol..

[68] Marx S.P., Winkler S., Hartmeier W. (2000). Metabolization of β-(2,6)-linked fructose-oligosaccharides by different bifidobacteria. FEMS Microbiol. Lett..

